# Home food procurement impacts food security and diet quality during COVID-19

**DOI:** 10.1186/s12889-021-10960-0

**Published:** 2021-05-19

**Authors:** Meredith T. Niles, Kristen Brassard Wirkkala, Emily H. Belarmino, Farryl Bertmann

**Affiliations:** 1 Department of Nutrition and Food Sciences and Food Systems Program, University of Vermont, Burlington, VT 05405 USA; 2 Gund Institute for Environment, University of Vermont, Burlington, VT 05405 USA

**Keywords:** Gardening, Hunting, Fishing, Foraging, Fruit and vegetable intake, Food insecurity, COVID-19, Diet quality, Red meat intake

## Abstract

**Background:**

Home food procurement (HFP) (i.e. gardening, fishing, foraging, hunting, backyard livestock and canning) have historically been important ways that people obtain food. Recently, some HFP activities have grown (e.g. gardening), while other activities (e.g. hunting) have become less common in the United States. Anecdotally, COVID-19 has sparked an increase in HFP evidenced by increased hunting licenses and shortages in seeds and canning supplies. HFP may have positive benefits for food security and diet quality, though research beyond gardening is especially limited in high-income countries.

**Methods:**

We examine HFP activities since the COVID-19 pandemic began, and their relationship to food security and dietary quality using multivariable logit models and matching analysis with a statewide representative survey (*n* = 600) of residents of Vermont, United States.

**Results:**

We find 29% of respondent households classified as food insecure since COVID-19, and higher prevalence of food insecurity among those experiencing a negative job change since COVID-19, households earning less than $50,000 annually, Hispanic and multi-race respondents. Nearly 35% of respondents engaged in HFP activities since the COVID-19 pandemic began; the majority of those gardened, and more than half pursued HFP activities more intensely than before the pandemic or for the first time. Food insecure households were more likely to pursue HFP more intensely, including more gardening, fishing, foraging, and hunting. Respondents who were food insecure, Black, Indigenous, People of Color, those with a negative job disruption, and larger households all had greater odds of increased intensity of HFP during the COVID-19 pandemic. HFP was significantly associated with eating greater amounts of fruits and vegetables; however, this effect was only significant for food secure households.

**Conclusion:**

Overall, these results suggest that HFP activities have increased since the start of the COVID-19 pandemic, and may be an important safety net for food insecure households. However, HFP for food insecure households does not translate into the same higher fruit and vegetable intake as found among food secure HFP households, suggesting this population may be trying to maintain intake, or that they may have potential important resource or technical assistance needs. Long-term, HFP activities may have important food security and diet quality impacts, as well as conservation implications, which should be more thoroughly explored. Regardless, the increased interest and intensity of HFP demonstrates opportunities for educational and outreach efforts.

**Supplementary Information:**

The online version contains supplementary material available at 10.1186/s12889-021-10960-0.

## Background

The COVID-19 pandemic has highlighted the uncertainty and fragility of food security and food access globally. In the United States, unemployment rates reached unprecedented levels at their height in April 2020, causing concerns among many Americans about how to access affordable and high-quality food [1]. Existing evidence suggests that home food procurement (i.e. backyard livestock, fishing, foraging, gardening, hunting, and canning, hereafter referred to as HFP) may offer opportunities to improve food security and diet quality (e.g. [2, 3]). HFP activities have varying levels of participation in recent decades. While homesteading [4] and backyard livestock, especially chickens, have become more fashionable in recent years [5], hunting has been declining for decades [6, 7]. However, since the COVID-19 pandemic began, there have been a number of stories from popular media outlets in the United States discussing a comeback of “victory gardens” in response to the pandemic [8, 9], increased interest and demand for hunting and fishing [10], and a shortage of canning supplies [11]. As well, previous research has found that depictions of wild food foraging in the media change in times of economic hardship from being discussed as more of a luxury to being conceptualized as a way to provide for basic needs [12]. Public discussion and interest around HFP practices seem to be shifting with COVID-19, but who is participating and what relationship do these activities have to food security and dietary outcomes? This study explores changes in HFP since the onset of the COVID-19 pandemic, and its relationship to food security and diet quality outcomes during the pandemic in a high-income country context.

The potential for HFP to improve food security and dietary outcomes has links to other challenging times, including in historical moments such as World War 2. At that time, planting “victory gardens” were patriotic acts to grow local food amidst disrupted supply chains [13]. It is estimated that 40% of the nation’s fruits and vegetables were produced via victory gardens during the war, demonstrating the potential for HFP to address food security challenges. But the current COVID-19 context has created new difficulties and significant increases in food insecurity in many countries, including the United States (e.g. [14, 15]). Nevertheless, existing evidence suggests that HFP may positively affect both food security and dietary quality outcomes in high-income countries through multiple pathways.

Evidence suggests that growing your own food contributes directly toward food availability and access. Taylor & Lovell [2] found that, while gardeners did not grow enough to sustain their families, 1/3 grew a substantial quantity and were self-sufficient in providing some items for a certain period of time during the growing season and almost all of these households said they always had enough to eat. Corrigan’s [16] interviews of five gardeners in Baltimore found that most perceived that they saved money from their gardens and that it allowed them to grow quality, fresh produce that otherwise may not have been accessible. They also found that many gardeners canned or froze their excess produce, allowing them access to these foods into the winter. These results may also translate beyond gardening to other food procurement practices, although research is even more limited in these areas. Smith et al. [17] found that participants from one reservation who participated in the Food Distribution Program on Indian Reservations who also hunted, fished, or foraged were more food secure than those who did not. Additionally, those who engaged in more than one practice were more secure than those who did only one. A survey of Canadian Inuit also found that households with an active hunter were more food secure than those without an active hunter [18]. Cooke et al. [19] found that many anglers in the United States often consume what they catch, with an average of 4700 g of edible fish provided through fishing annually, even if their original motivation for fishing is recreation. As well, African American anglers are more likely to consider fishing important for providing food, compared to non-African American anglers [20] and more likely to keep fish that they have caught [21], though these studies did not examine food security outcomes. This direct food procurement may also lead to cost savings realized by not purchasing food, which enable money to be available for the purchase of other foods, or for other financial priorities.

Realized cost savings from HFP may be another factor linking HFP to better food security outcomes. Perceived cost savings does appear to be a common motivation for those producing their own food [17, 22] and there have been a number of studies suggesting that this may in fact be the case [2, 3, 23, 24]. Home gardeners in San Jose, California reported that cost savings of gardening allowed them to eat produce they otherwise would not have had access to [25]. However, many studies looking at cost savings were analyzing the results of nonprofit programs in which gardeners were supported with resources to help set up their gardens, and therefore had a smaller up-front investment, which could have impacts on food security outcomes. Csortan et al. [26] found that 65% of South Australian home food gardeners surveyed would break-even on garden investments in five years or less and then start saving money. In such a case, gardening would not be a sufficient means for achieving food security in the short-term in response to an economic crisis. They also found that the number of years of gardening experience appeared to have a positive impact on productivity and resource efficiency, leading to additional concerns for new gardeners [26].

In addition to the potential for cost savings and increased food security, HFP may lead to a higher quality, more diverse diet, including one that may be more culturally appropriate [27, 28]. Growing one’s own produce is linked to increased fruit and vegetable intake [3, 25, 29–31]. Hunting, fishing, and foraging may also lead to a more nutritious and diverse diet; for example, 80% of people surveyed on a native reservation said that hunting, fishing, and foraging made their diets more diverse and 72% said these practices improved the quality of their diet [17]. Stark et al. [32] found that wild edible greens were abundant in three low-income neighborhoods in California, and offered potential nutrient density comparable to some common nutritious vegetables, such as kale. Some research suggests that growing one’s own food may also lead to improved nutritional knowledge [33, 34] and changes in eating habits for the long-term [24, 34, 35]. This may also be true of children, who are more likely to try vegetables when they garden [36].

Prior research suggests that partaking in HFP strategies may lead to an increase in food security and diet quality outcomes, but the current research is limited, especially as it pertains to the impact of hunting, fishing, and foraging practices in high-income countries. Further, COVID-19 has changed the way many people work, live, and shop, potentially providing opportunities or new barriers to HFP and new challenges for food security and high-quality diets. Emerging evidence indicates that dietary quality has decreased during the COVID-19 pandemic in many places (e.g. [37, 38], offering potential opportunities for HFP to counter such trends. Existing evidence of HFP activities since the start of the COVID-19 pandemic is limited, though our previous work found about half of respondents reported producing, foraging, hunting, or canning last year and nearly one third were engaging in those activities at the time of the survey [39]. Chenarides et al. [40] examined urban gardening before and during the COVID-19 pandemic, finding lower participation in community gardens as compared to at-home gardens. Constant et al. [38] found having a garden/terrace positively associated with unhealthy behaviors including eating fewer fruits and vegetables during the COVID-19 lockdown in France. Finally, though a few commentaries have discussed the potential benefits of home gardens during COVID-19 (e.g. [41, 42]), to our knowledge, no population-based studies have comprehensively assessed HFP activities during the pandemic and its relationship to food security and diet quality outcomes. This study aims to fill this gap by surveying a representative sample of people in Vermont, a rural US state, to understand their HFP strategies, change in activity during the first 5 months of the COVID-19 pandemic, and the relationship of HFP to food security and diet quality. In a predominantly rural state such as Vermont, these concerns are especially pressing, as rural areas are estimated to have 50% higher rates of food insecurity than urban areas [43].

## Methods

### Survey development and sampling strategy

The data were collected using a survey instrument developed initially in March 2020 [44], in collaboration with other researchers as part of the National Food Access and COVID research Team (NFACT) [45]. The survey was further refined [46], with the latter forming the basis for this data collection. The survey measures multiple components of food access, food security, dietary intake, home food procurement, COVID-19 experiences and food assistance program participation, as well as individual and household sociodemographics. Institutional Review Board approval was obtained from The University of Vermont (IRB protocol 00000873) prior to any data collection. The survey utilizes validated measurements when possible (Table 1), and was also validated prior to release of Version 1 in Vermont with 25 eligible (18 and over) respondents using Cronbach alpha and factor analysis [14]. All question sets obtained an internal validity of alpha > 0.70 [47, 48].

**Table 1:** Complete list of variables, questions and measurement utilized in this analysis

Variable Name	Question/Scale	Measurement
Food Secure	6 item food security module from USDA	1 = Food Secure (0 or 1 affirmatives in module), 0 = Food Insecure (Affirmative to 2 or more questions in module)
**Dietary Quality Variables:**		
Fruit Intake	About how many cups of fruit (including 100% pure fruit juice) do you eat or drink each day? Examples of 1 cup for fruit include 1 small apple, 1 large banana, 1 cup (8 oz.) of 100% juice or canned fruit, or ½ cup of dried fruit.	0 = None, 1 = ½ cup or less, 2 = ½ to 1 cup, 3 = 1–2 cups, 4 = 2–3 cups, 5 = 3–4 cups, 6 = 4 cups or more
Vegetable Intake	About how many cups of vegetables (including 100% vegetable juice) do you eat or drink each day? Examples of 1 cup of vegetables include 1 cup of cooked leafy greens, 2 cups of lettuce or raw greens, 12 baby carrots, 1 medium potato, or 1 large raw tomato.	
Red Meat Intake	How often did you eat red meat (such as beef, pork, ham, sausage, veal lamb)? Do not include chicken, turkey or seafood. Include red meat you had in sandwiches, lasagna, stew, and other mixtures.	0 = Never, 1 = 1 time last month, 2 = 2–3 times last month, 3 = 1 time per week, 4 = 2 times per week, 5 = 3–4 times per week, 6 = 5–6 times per week, 7 = 1 time per day, 8 = 2 or more times per day
Processed Meat Intake	How often did you eat any processed meat, such as bacon, lunch meats, or hot dogs? Include processed meats you had in sandwiches, soups, pizza, casseroles, and other mixtures. Processed meats are those preserved by smoking, curing, or salting, or by the addition of preservatives.	
Fruit/Vegetable Change	I have been eating more, less, or about the same amount of fruits and vegetables per day.	1 = Less, 2 = Same, 3 = More
Red/Processed Meat Change	I have been eating more, less, or about the same amount of processed meat, lunch meats, and red meats.	
**Home Food Procurement Variables:**		
COVID-19 HFP (HFP)	Indicated that the household accessed local food through gardening, fishing, foraging, hunting, backyard livestock or using your own canned good at any points since the COVID-19 pandemic began	Binary variable (1 = home food procurement activity, 0 = no activity)
Garden Since	Respondent that has gardened since COVID-19	1 = Had a garden since COVID-19, 0 = No garden since COVID-19
Fishing Since	Respondent that has fished since COVID-19	1 = Fished since COVID-19, 0 = No fishing since COVID-19
Foraging Since	Respondent that has foraged since COVID-19	1 = Foraged since COVID-19, 0 = No foraging since COVID-19
Hunting Since	Respondent that has hunted since COVID-19	1 = Hunted since COVID-19, 0 = No hunting since COVID-19
Livestock Since	Respondent that has backyard livestock since COVID-19	1 = Had backyard livestock since COVID-19, 0 = No backyard livestock since COVID-19
Canning Since	Respondent that has used own canned goods since COVID-19	1 = Used own canned goods since COVID-19, 0 = No canning since COVID-19
HFP More	Subset of respondents that pursued HFP- Any respondent that indicated they pursued a HFP activity “for the first time this year”, or “I have previously done this, but did it more this year”	Binary variable (1 = more intense HFP, 0 = no change in activity, or pursued less this year)
Gardens More	Any respondent that indicated they pursued gardening “for the first time this year”, or “I have previously done this, but did it more this year”	1 = More intense or new, 0 = same or less than before
Fishing More	Any respondent that indicated they pursued fishing “for the first time this year”, or “I have previously done this, but did it more this year”	
Foraging More	Any respondent that indicated they pursued foraging “for the first time this year”, or “I have previously done this, but did it more this year”	
Hunting More	Any respondent that indicated they pursued hunting “for the first time this year”, or “I have previously done this, but did it more this year”	
Livestock More	Any respondent that indicated they pursued backyard livestock “for the first time this year”, or “I have previously done this, but did it more this year”	
Canning More	Any respondent that indicated they pursued canning “for the first time this year”, or “I have previously done this, but did it more this year”	
**Demographic Variables:**		
Female	Which of the following best describes your gender identity?	1 = Female, 0 = Male
Children in HH	How many people in the following age groups currently live in your household (including you)? Household includes people currently living within your home, including family and non-family members.	1 = Any children in household, 0 = No children in household
Over 55	Please select your age group	1 = Respondent 55 or older, 0 = Respondent 55 or younger
Race/Ethnicity (BIPOC/and or/Hispanic)*	What is your race? Are you of Hispanic, Latino, or Spanish origin?	1 = Respondent identify as Asian, Black or African America, Native America, White, Mixed Race, and/or Hispanic, Latino or Spanish origin, 0 = Respondent identifies as white and non-Hispanic, Latino or Spanish origin
Negative Job Change	Have you or anyone in your household experienced a loss of income or job since the COVID-19 outbreak (March 11th)?	1 = Any job change (job loss, reduced hours or income at job, furloughed), 0 = No job change
Less $50 K	Which of the following best describes your household income range in 2019 before taxes?	1 = Household income below $50,000 a year, 0 = Household income above $50,000 a year
HH Size	How many people in the following age groups currently live in your household (including you)? Household includes people currently living within your home, including family and non-family members.	1 = 1 person, 2 = 2 people, 3 = 3 people, 4 = 4 people, 5 = 5 people, 6 = 6 people, 7 = 7 people or more

* We would like to acknowledge we aggregate this data because of the low number of respondents identifying as BIPOC and/or Hispanic. While this survey is representative of Vermont state characteristics on race and ethnicity, the sample size is too low to analyze racial and ethnic groups in a disaggregated format in models. We have disaggregated race and ethnicity in reporting food security statistics in the results, but aggregate race and ethnicity together for modeling and matching

Participants were recruited through an online survey administered by Qualitrics (Provo, UT), using a general population sample representative to the state of Vermont with respect to income, race and ethnicity. This sample was achieved by matching sample recruitment quotas to the income, race (White, Black or African American, American Indian and Alaska Native, Asian, Native Hawaiian or Other Pacific Islander, and Two or more races), and ethnicity (Hispanic, non-Hispanic) population profile of Vermont in the American Community Survey [49]. A total of 600 people ages 18 and over responded to the survey, representing a margin of error (95% confidence level) for this segment of the Vermont population of +/− 4% [50]. The survey was administered in August and September 2020 and received a response rate of 35%.

### Variables of interest

We explore three self-reported dependent variables in this analysis (Table 1). First, food security status, as measured through the US Department of Agriculture 6-item short-form food security module [51] where food insecurity is classified as answering affirmatively to two or more out of six questions. This was modified to ask respondents about food security since the start of the COVID-19 pandemic (approximately 5 months at the time of the survey) rather than the traditional 12-month period. Second, current fruit and vegetable intake was measured through the National Cancer Institute’s 2-item screener [52], which was modified to apply to the last month and some example foods were removed to shorten it. Current red and processed meat intake was measured using two questions from the Dietary Screener Questionnaire in the National Health and Nutrition Examination Survey (NHANES) 2009–10. Finally, we developed new questions to measure perceived change in fruit/vegetable and red meat/processed meat consumption since the onset of the COVID-19 pandemic. Independent variables included multiple questions related to previous and current HFP, specific HFP activities, and changes in HFP activities during the COVID-19 pandemic, as well as several household and individual-level demographics (Table 1).

### Statistical analysis

We utilize a series of logistical regression models, reporting with odds ratios to examine how demographic factors correlate with home food procurement since the COVID-19 pandemic began, and the different HFP strategies. We use chi-square tests to examine food security and diet quality changes since the start of the COVID-19 pandemic as it relates to HFP, specific HFP activities, and intensity of HFP. We use one-way analysis of variance (ANOVA) to examine diet quality intake at the time of the survey as it relates to HFP, specific HFP activities, and intensity of HFP. Then, to examine how HFP, intensity of HFP, and specific HFP activities relate to both food security outcomes and dietary quality, we use nearest neighbors matching techniques. We report statistical significance as anything *p* < 0.05.

Matching techniques are useful with observational data to estimate causal effects of treated and control groups, aiming to balance the distribution of covariates across treated and control groups [53]. Here we explore how HFP, intensity of HFP, or specific HFP activities are “treatments” on food security and diet quality, using demographic factors as matching covariates across groups. We use six demographic covariates in our matching analysis: female, children in household (HH), race/ethnicity (Black, Indigenous, People of Color (BIPOC)/and or Hispanic), negative job change, household income less than $50,000 (less $50 k), and HH size (Table 1), which are likely to be associated with the treatment and outcome [54, 55]. Matching techniques also require defining a distance (measure of similarity between the individuals). We use a nearest neighbor matching approach with a Mahalanobis distance, which accounts for covariance among variables, and is documented to work well with fewer than eight covariates [56, 57]. For each treated individual, nearest neighbor matching selects a control individual with the smallest distance from that individual. For example, if we are exploring HFP, the technique would have people who did and did not engage in HFP as “treatment” and control groups, and then match a treatment and control respondent together based on similar demographic covariates included in the analysis (e.g. household size and job change status). In all our models we use nearest neighbor matching with between three and five matches per observation, meaning each observation was matched with at least three closest other observations within the control and treatment groups. Since we are interested in the difference between expected outcomes among those with and without “treatment” (HFP), we report average treatment effect on the treated, and ensure the existence of potential matches in the control group to satisfy the common support condition [58]. We report the total number of matched individuals for each matching outcome in results tables to confirm the existence of matches for all treatments. Furthermore, we implement a maximum caliper of 0.1 for all matching analyses with the exception of matching involving “more” HFP since COVID-19, where we implement a caliper of 0.3 because of a smaller sample size. The implementation of these calipers satisfies the overlap and common support requirements, and ensures high quality matching [58].

## Results

### Respondent characteristics

Table 2 details the specific respondent characteristics, which reflect the demographic composition of the Vermont population for the gender, race, and income distribution. Overall, 67.3% of the respondents were female (std. dev = 0.47), and 30.2% of respondents had children in the household (std. dev = 0.46). Forty-four percent of respondents were age 55 years or older. Reflecting the racial/ethnic profile of Vermont, 8.3% of respondents identified as BIPOC and/or Hispanic ethnicity (std. dev = 0.28). More than 46% of respondents lived in a household that had experienced a negative job change during the first 5 months of the COVID-19 pandemic (job loss, loss of income or hours from job, or furlough) (std. dev = 0.50). Household size was on average 2.57 (std. dev = 1.34), with 60.2% of households with 2 or fewer people.

**Table:** Table 2

Characteristic	Respondents (***N*** = 600)
Age - no. (%)	
18–34	153 (25.5)
35–54	182 (30.3)
55+	263 (43.8)
Children in household - no. (%)	
Yes	178 (30.2)
No	415 (70.0)
Gender - no. (%)	
Female	404 (67.3)
Male	190 (31.7)
Transgender/Non-binary/Self-Described	6 (1.0)
BIPOC -Race - no. (%)	
White	559 (93.2)
Two or more races	22 (3.7)
American Indian or Alaska Native	5 (0.8)
Asian	4 (0.7)
Black or African American	9 (1.5)
BIPOC - Ethnicity - no. (%)	
Not Hispanic or Latino	583 (97.2)
Hispanic or Latino	17 (2.8)
2019 Household Income - no. (%)	
Less than $10,000 per year	39 (6.5)
$10.000–$24,999	81(13.5)
$25,000–$49,999	141 (23.5)
$50,000–$74,999	110 (18.3)
$75,000 - $99,999	77 (12.8)
$100,000 or more	145 (24.1)
Job change during the COVID-19 pandemic - no. (%)	
Lost job	149 (24.8)
Reduced hours or income	208 (34.7)
Furloughed	122 (20.3)
Any change	270 (46.2)
No changes	314 (53.8)
Household Size - no. (%)	
1 to 2	357 (60.2)
3 to 5	211 (35.6)
6 or more	25 (4.2)

### Descriptive statistics of key variables

**Fig. 1:**
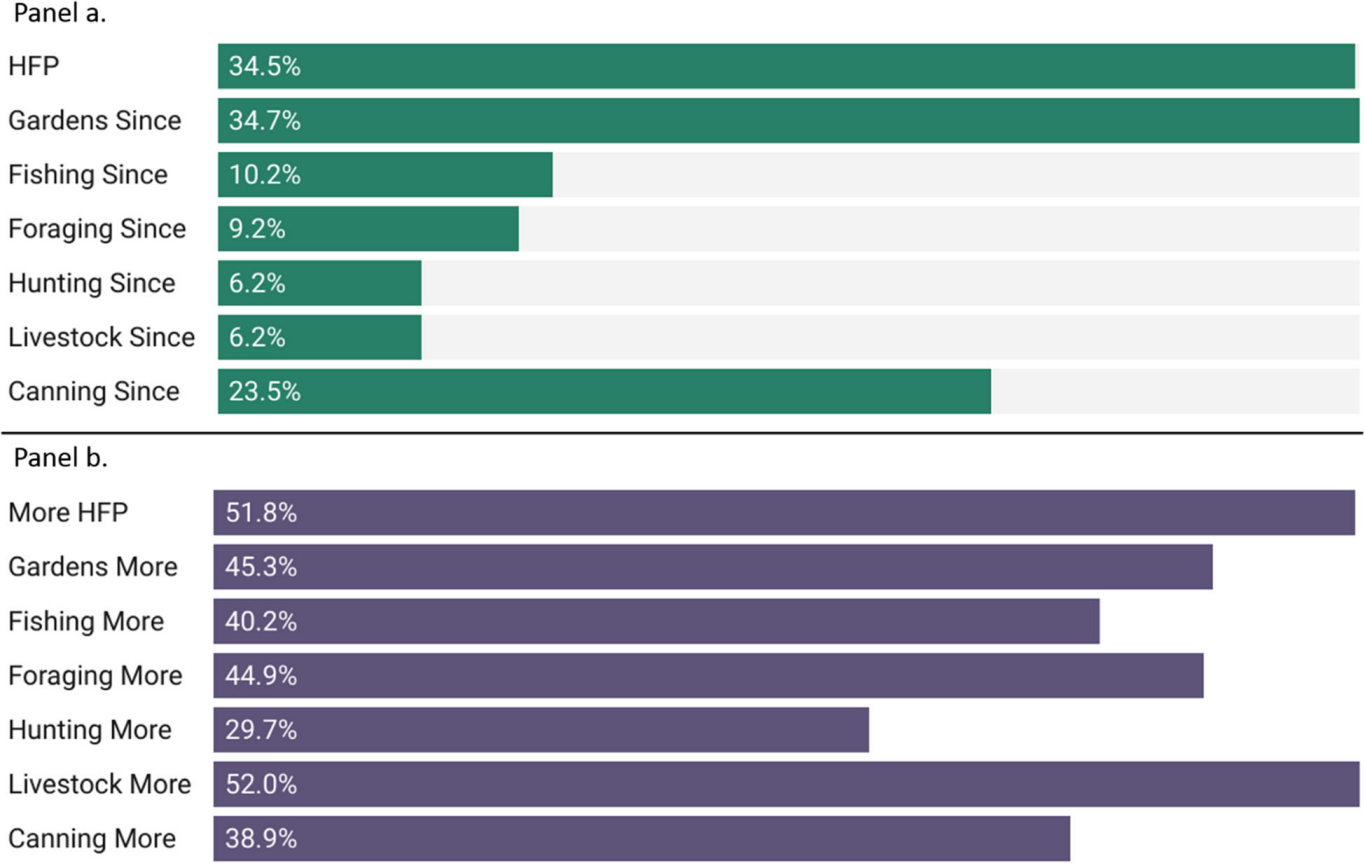
**a** Percent of respondents engaging in any HFP, and specific HFP activities since COVID-19. Percentages include all respondents (*n* = 600). **b** Among respondents who engaged in any HFP (*n* = 205), percent of those that increased intensity or did a new HFP activity since COVID-19

Among all respondents, 34.5% (*n* = 205) engaged in HFP activity during the first 6 months of the COVID-19 pandemic, with the greatest number of respondents gardening (34.7%), followed by canning (23.5%) and fishing (10.2%) (Fig. 1). Among respondents who engaged in HFP, 51.8% (*n* = 128) did at least one HFP activity more intensely since the COVID-19 pandemic began or for the first time during the COVID-19 pandemic, with the greatest increase in intensity of activity among backyard livestock (52%, *n* = 26), gardening (45.3%, *n* = 106), and foraging (44.9%, *n* = 31).

On average, respondents self-reported they ate between 1 and 2 cups cumulatively of fruit (mean = 2.20) and vegetables (mean = 2.74) per day, though 11 and 5% of respondents ate no fruit or vegetables respectively daily. Respondents self-reported they ate red meat (mean = 3.34) and processed meat (mean = 3.15) about one time per week, with 10% each indicating they never eat red or processed meat. Nearly one in four (23.3%) respondents indicated eating less fruits and vegetables during the pandemic as compared to before, 65.5% reported eating the same as before COVID-19, and 11.2% reported eating more. Changes in red and/or processed meat consumption were also indicated by about one-third of respondents, with 25.9% eating less red and/or processed meat since the start of the COVID-19 pandemic, and 7.9% eating more.

### Demographics of food security

Among our dependent variables, 71% (*n* = 414) of respondent households were classified as food secure since COVID-19 (29% food insecure, *n* = 169). To assess the relationship of our demographic controls on food security, we ran a multivariable logit model (Supplementary Table 1). Respondents 55 and over were at higher odds of food security (OR = 2.52, *p* = 0.001), while households experiencing a negative job disruption (OR = 0.47, *p* = 0.001), and those earning less than $50K annually (OR = 0.134, *p* < 0.001), were at reduced odds of food security. Disaggregating race and ethnicity demonstrates lower rates of food security among Black (50%), Hispanic (50%), and multiple race respondents (66.6%); however, these results are not statistically significant (*p* < 0.05) with a chi-square test, likely because of our low sample size (Supplementary Table 2).

### Demographics of home food procurement

Using a multivariate logit model, we examine how demographics correlate with different aspects of HFP. We find that households experiencing a negative job change have 1.53 greater odds (*p* = 0.022) of HFP since the COVID-19 pandemic (Table 3). Among those that did HFP since COVID-19, we find that multiple demographic factors are correlated with increased intensity of HFP during the pandemic. Specially, BIPOC /Hispanic respondents (OR = 3.58, *p* = 0.026), households experiencing a negative job change (OR = 1.89, *p* = 0.026), and larger households (OR = 1.48, *p* = 0.021) were at significantly greater odds of increased intensity of HFP while respondents over 55 were at significantly reduced odds of increasing intensity during the pandemic (OR = 0.49, *p* = 0.029) (Table 4).

**Table 3:** Multivariate logit model predicting COVID-19 home food procurement (HFP) activities with demographics

Variable	Odds Ratio	Std. Error	P=	95% Confidence Interval	
Female	0.929	0.181	0.704	0.634	1.360
Children in HH	1.074	0.288	0.789	0.636	1.816
Over 55	1.299	0.279	0.223	0.853	1.979
BIPOC/Hispanic	1.082	0.351	0.807	0.573	2.043
Negative Job Change	1.525	0.282	0.022	1.062	2.191
Less $50 K	0.756	0.141	0.133	0.525	1.089
HH Size	0.918	0.084	0.351	0.767	1.099

**Table 4:** Multivariate logit model predicting increased intensity of HFP since COVID-19 with demographics

Variable	Odds Ratio	Std. Error	P=	95% Confidence Interval	
Female	1.436	0.450	0.249	0.777	2.655
Children in HH	0.758	0.322	0.514	0.329	1.745
Over 55	0.486	0.160	0.029	0.255	0.927
BIPOC	3.585	2.051	0.026	1.169	11.000
Negative Job Change	1.894	0.543	0.026	1.080	3.320
Less $50 K	0.855	0.254	0.599	0.478	1.532
HH Size	1.477	0.249	0.021	1.062	2.054

Multivariate logistical regression models predicting the specific types of all six HFP activities since the COVID-19 pandemic by demographics found multiple significant factors. Respondents with a negative job change were at increased odds of gardening (OR = 1.43, *p* = 0.055), while households making less than $50,000 annually were at reduced odds (0.63, *p* = 0.014). Respondents over 55 were at reduced odds of fishing since the start of the COVID-19 pandemic (OR = 0.50, *p* = 0.051), while respondents with a negative job change (OR = 2.13, *p* = 0.014) were at increased odds. Women were at reduced odds of hunting during the pandemic (OR = 0.46, *p* = 0.034. Respondents over 55 were at reduced odds of having backyard livestock during the pandemic (OR = 0.16, *p* = 0.001) (Supplementary Tables 3–8).

### Home food procurement and food security

**Fig. 2:**
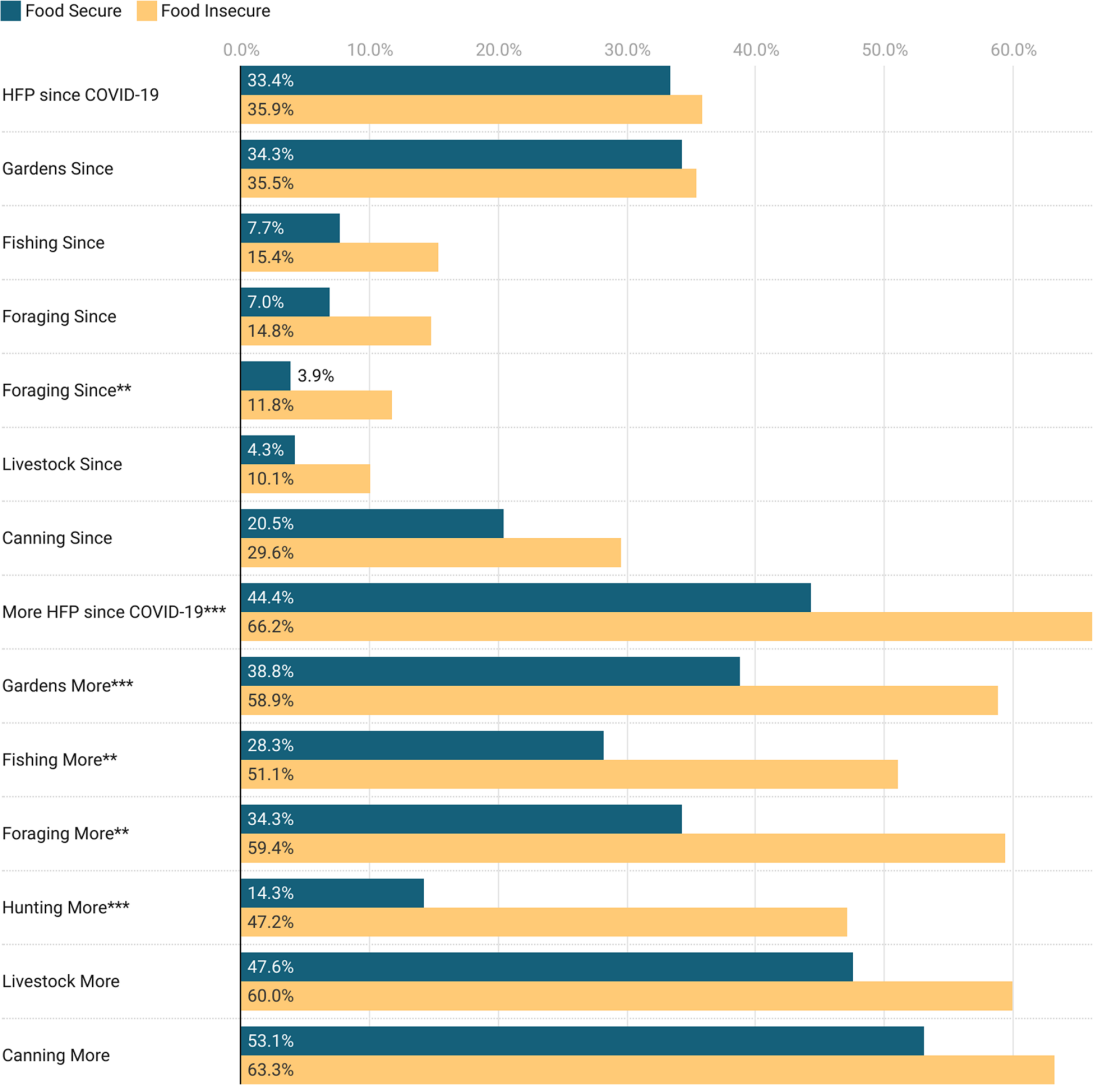
Percent of food secure and food insecure households engaging in various types of HFP activities and intensity since COVID-19. (** = *p* < 0.05, *** = *p* < 0.01, Supplementary Table 9). Questions about any HFP, and specific HFP since COVID-19 include all respondents. Questions about increased HFP activity are asked only of respondents engaging in HFP (*n* = 250)

At the aggregate, we do not find statistically significant differences in engagement in HFP between food secure and insecure households. However, we do find that food insecure households are significantly more likely to engage in certain types of HFP activities. Overall, food insecure respondents were significantly more likely to be fishing (*p* = 0.005), foraging (*p* = 0.003), hunting (*p* < 0.001), canning (*p* = 0.019), and have backyard livestock (*p* = 0.008) during the COVID-19 pandemic (Fig. 2).

We use matching approaches to examine the effect of HFP since the COVID-19 pandemic on household food security. We find no association between HFP since COVID-19 and food security while controlling for multiple demographic factors. However, exploring the effect of specific HFP activities during the pandemic on food security outcomes, we find that fishing (b = − 0.174, *p* = 0.007), hunting (b = − 0.297, *p* < 0.001), and canning (b = − 0.149, *p* = 0.001) are all negatively associated with food security (Table 5).

**Table 5:** Food security outcomes as related to HFP using nearest neighbors matching analysis. Each row indicates a separate matching analysis, where the HFP variable was used as a “treatment” while using six demographic controls (Female, Children in HH, BIPOC, Negative Job Change, Less $50 k, HH size) to conduct the matching. Negative coefficients indicate an association with reduced food security

	Coefficient	Robust Std. Error	p=	95% Confidence Interval		Treated *n* = (Matched n=)	Control *n* = (Matched n)
HFP Since COVID	−0.02	0.041	0.627	−0.102	0.061	193 (193)	355 (193)
Garden Since	−0.055	0.038	0.146	−0.130	0.193	197 (197)	357 (197)
Fishing Since	−0.174	0.064	0.007	−0.301	−0.048	56 (56)	498 (56)
Foraging Since	−0.112	0.078	0.151	−0.265	0.041	53 (53)	501 (53)
Hunting Since	−0.297	0.082	0.000	−0.458	−0.137	34 (34)	520 (34)
Livestock Since	−0.144	0.086	0.093	−0.311	0.024	35 (35)	519 (35)
Canning Since	−0.149	0.045	0.001	−0.237	− 0.062	133 (133)	421 (133)
HFP More	−0.206	0.055	0.000	−0.314	− 0.098	117 (117)	113 (117)
Gardens More	−0.202	0.057	0.000	−0.315	− 0.089	98 (98)	120 (98)
Fishing More	−0.241	0.109	0.027	−0.455	− 0.027	36 (36)	53 (36)
Foraging More	− 0.130	0.133	0.327	− 0.390	0.130	30 (30)	35 (30)
Hunting More	−0.225	0.176	0.201	−0.570	0.120	20 (20)	48 (20)
Livestock More	−0.202	0.750	0.007	−0.350	−0.055	25 (25)	20 (25)
Canning More	−0.318	0.073	0.000	−0.462	−0.175	58 (58)	89 (58)

We also find through chi-square analysis, significant associations between food security and intensity of HFP since the COVID-19 pandemic began, with 66.2% of food insecure households increasing intensity of HFP since the COVID-19 pandemic began, compared to 44.4% of food secure households (*p* = 0.002). Food insecure households were also more likely to engage in HFP more intensely since COVID-19, and do certain activities more (*p* < 0.050) (Fig. 2). Matching results with demographic controls confirm that engaging in HFP more overall as well as more intensely gardening, fishing, and canning since the COVID-19 pandemic were associated with reduced food security (*p* < 0.050, Table 5).

### Home food procurement and diet quality

**Fig. 3:**
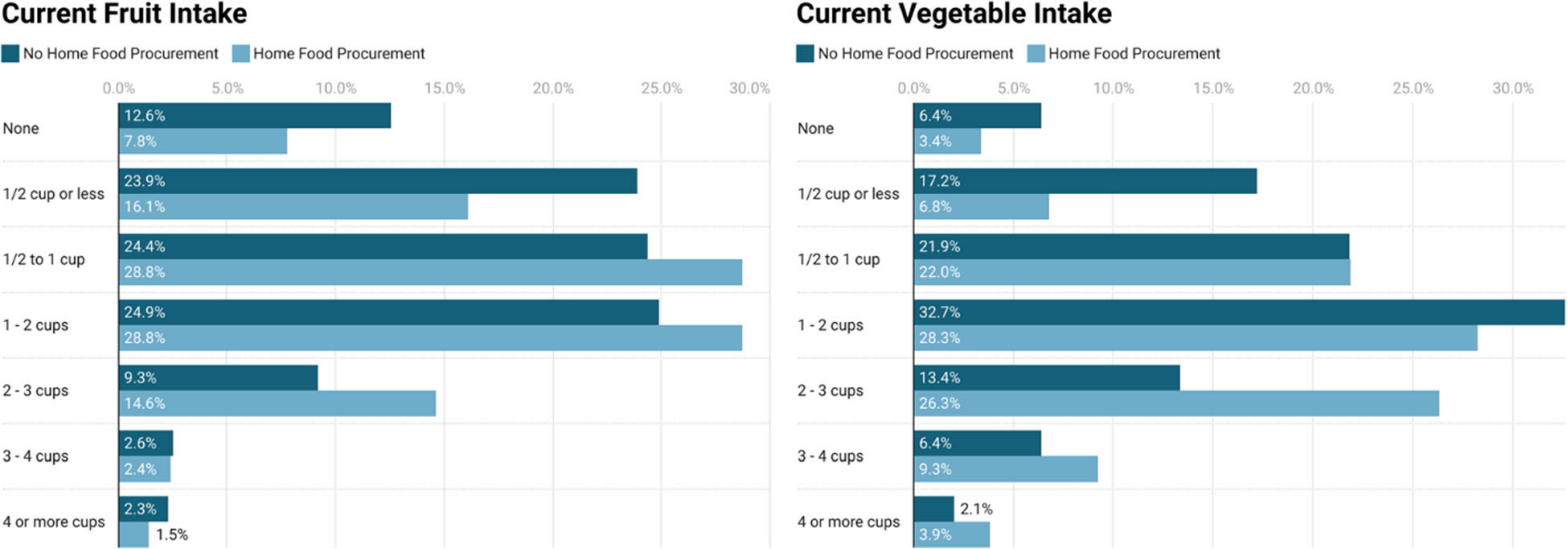
Current self-reported vegetable and fruit intake among respondents engaged or not in HFP since the COVID-19 pandemic. On average, respondents who engaged in HFP are significantly more likely to be eating more fruit (*p* < 0.05) and vegetables (*p* < 0.001). However, these results differed by a household’s food security status (Table 6)

We use ANOVA to examine the current dietary quality at the time of the survey as it relates to HFP, specific HFP activities and intensity of HFP. Overall, respondents engaging in HFP were significantly more likely to eat greater amounts of fruits (mean 2.40 cup equivalents compared to 2.11, *p* = 0.02) and vegetables (mean 3.11 cup equivalents compared to 2.57, *p* < 0.001) (Fig. 3).

We find no significant differences between HFP and intake of red meat (mean = 3.43 compared to 3.33, *p* = 0.490) or processed meat (mean = 2.98 compared to 3.24, *p* = 0.100). Using matching techniques, with demographic controls, we examine current fruit, vegetable, red meat and processed meat intake as it relates to HFP, increased HFP, and relevant specific HFP activities (i.e. gardening, foraging and canning for fruit and vegetable intake and fishing, hunting and backyard livestock for red and processed meat intake). We find the “treatment” of HFP to have a significant and positive relationship to higher fruit (b = 0.292, *p* = 0.019) and vegetable intake (b = 0.487, *p* < 0.001) (Supplementary Tables 10 and 11). We find no relationship between red meat intake and HFP (Supplementary Table 12), but we do find HFP since the COVID-19 pandemic associated with reduced processed meat consumption (b = − 0.365, *p* = 0.025) (Supplementary Table 13). Further, we also assess the relationship of HFP engagement to dietary outcomes specifically among food secure and food insecure households, which yields different results (Table 6). Importantly, HFP among food secure households is associated with higher fruit (b = 0.309, *p* = 0.022) and vegetable intake (b = 0.669, *p* < 0.001); however, among food insecure households, we see no significant effect of HFP on any dietary intake outcomes. This suggests that the “treatment” of HFP is significant for food secure households and fruit and vegetable intake, but not for food insecure households.

**Table 6:** Dietary intake outcomes as related to HFP among food secure and food insecure households using nearest neighbors matching analysis. Each row indicates a separate matching analysis, where the HFP variable was used as a “treatment” while using six demographic controls (Female, Children in HH, BIPOC, Negative Job Change, Less $50 k, HH size) to conduct the matching. Negative coefficients indicate an association with lower intake

	Coefficient	Robust Std. Error	p=	95% Confidence Interval		Treated ***n*** = (Matched n=)	Control ***n*** = (Matched n)
**Current Fruit Intake**							
HFP- Food Secure	0.309	0.135	0.022	0.045	0.573	201 (201)	364 (201)
HFP- Food Insecure	0.159	0.292	0.586	−0.413	0.732		
**Current Vegetable Intake**							
HFP- Food Secure	0.669	0.145	0.000	0.385	0.953	201 (201)	364 (201)
HFP- Food Insecure	−0.064	0.29	0.825	−0.633	0.504		
**Current Red Meat Intake**							
HFP- Food Secure	−0.018	0.200	0.926	−0.41	0.373	201 (201)	364 (201)
HFP- Food Insecure	0.065	0.432	0.88	−0.781	0.912		
**Current Processed Meat Intake**							
HFP- Food Secure	−0.266	0.187	0.154	−0.632	0.098	201 (201)	364 (201)
HFP- Food Insecure	0.219	0.364	0.548	−0.494	0.932		

Examining the effect of increasing intensity of HFP and specific HFP activities, we find that gardening and canning since the COVID-19 pandemic began have significant effects on higher current intake of fruits (gardening b = 0.392, p = 0.001, canning b = 0.275, *p* = 0.044) and vegetables (gardening b = 0.551,*p* < 0.001; canning b = 0.513, *p* < 0.001) (Supplementary Tables 10 and 11). We find no significant effect of increased intensity of HFP on current red or processed meat intake. We also find having backyard livestock (b = 1.020, *p* = 0.001) since the start of the COVID-19 pandemic is associated with higher current red meat intake (Supplementary Tables 12 and 13).

We use chi-square tests to examine the change in dietary quality outcomes since the COVID-19 pandemic began as it relates to HFP, specific HFP activities and intensity of HFP. We find households engaging in HFP have a higher proportion of respondents with increased fruit and vegetable intake (15.6% compared to 8.7%, *p* = 0.021) which is confirmed through matching techniques with demographic controls (b = 0.116, *p* = 0.029) (Supplementary Table 14). However, we find no other significant effects of increased intensity of HFP, or specific HFP activities on change in fruit and vegetable intake (Supplementary Table 14) or meat intake (Supplementary Table 15) since the start of the COVID-19 pandemic.

## Discussion

Overall, we find a significant increase in HFP since the beginning of the COVID-19 pandemic, evidence that has been documented in the popular media, but not yet widely shown through peer-reviewed literature. Those engaging in HFP were more likely to be in households with negative job changes, and increased intensity was more likely among those with negative job changes, BIPOC respondents, and larger households. While we do not find that food secure and insecure households engage in HFP at different levels overall, food insecure households are more likely to have increased intensity of HFP during the pandemic. Engagement in some types of HFP activities, as well as overall increasing intensity of some activities is also associated with reduced food security. Though we find that nearly 25% are eating less fruits and vegetables since before the onset of the COVID-19 pandemic, we also find that HFP is positively associated with higher fruit and vegetable intake; however, this effect is only statistically significant among food secure households engaging in HFP, not food insecure households engaging in HFP. These results were especially prevalent among gardening and canning households, while red meat intake was higher among households with backyard livestock.

These results have several important implications. First, they suggest that food insecure households engage in HFP as a potential coping mechanism for food insecurity, and this appears to have been especially true during the first US growing season during the COVID-19 pandemic. This is further corroborated by the results that those with negative job changes were also more likely to be engaging in HFP and increasing the intensity of their engagement. More than 2/3 of food insecure households engaged more intensely in HFP or for the first time during the first five months of the pandemic. It is also important to note that a higher percentage of food insecure households are engaging more in non-gardening HFP activities (e.g. hunting, fishing, foraging) during the pandemic. Coupled together, these results provide important evidence about the reliance on HFP during a pandemic, and as a “safety net” for many potential households engaging in these activities for the first time or more intensely than before.

Our results are counter to some of the existing research that demonstrates that households using HFP are more food secure than those not using HFP [17], though the existing research on this topic is limited. There are several potential explanations for these different findings. First, the existing research in a Western context generally has had small sample sizes (e.g. [16]), and often focused on specific populations such as Native Americans [17]. This larger sample may provide additional insight into how food insecure households rely on HFP to minimize or lessen their food insecurity in new ways. Second, our analysis is specifically focused on the COVID-19 pandemic, an unprecedented time in recent history, in which unemployment and job loss, as well as food supply chain disruptions were widespread, triggering levels of food insecurity not seen in decades. Indeed, given that cost savings is often a motivation for HFP [3, 23, 24], such financial and lifestyle disruptions were likely an important component of HFP motivation and increased intensity. Finally, our study asked about a suite of HFP strategies, while other studies have typically focused on a single strategy such as gardening or fishing. This may be especially important when interpreting the results, since a larger percentage of food insecure households as compared to food secure households were engaging in non-gardening activities, which may have different potential impacts on food security. Hunting, fishing, and foraging for example, may not actually secure food in the same ways that gardening or backyard livestock could more reliably, at least during the time period in which our survey was conducted (e.g. summer before major hunting seasons).

Our results also demonstrate clear links between HFP and diet quality outcomes, especially for current fruit and vegetable intake among respondents using HFP, gardening and canning. These results confirm previous research findings that gardening is correlated with increased fruit and vegetable intake (e.g. [25, 29, 31]. However, this analysis goes further to demonstrate that these positive benefits are only found among food secure households, not food insecure households. This has important programmatic and policy implications, as it suggests that food insecure households may have less resources, time or capacity to engage in HFP in ways that may provide increased fruit and vegetable intake. Alternatively, these findings could suggest that food insecure households are using HFP as a strategy to maintain fruit and vegetable intake whereas food secure households are supplementing their usual intake with foods that they procure themselves. Since there was no significant difference in the percent of food secure versus food insecure households engaging in gardening or canning, the type of activity does not explain these differences. Further, food insecure households were engaging in gardening and foraging more intensely or for the first time as compared with food secure households since the start of the COVID-19 pandemic. This may signal that food insecure households are “new” to HFP, and may lack the necessary resources or capacity to engage in HFP activities. We suggest that this result should form the basis of further research, especially around the economic or other barriers that may exist for HFP engagement among food insecure households, and the use of HFP to replace vs. augment intake of nutrient-dense foods during economically challenging times.

Our work also demonstrates that food insecure households were significantly more likely to increase intensity of hunting since the COVID-19 pandemic, though this was not associated with a change in red meat intake. Interpreting the implications of these results (i.e. whether this is a positive or negative health outcome) is challenging, since prior research among a Native American population found that hunting, fishing and foraging increased the diversity and quality of diets [17]. While red meat intake is linked to various adverse health outcomes (e.g. [59–62]), not all red meats have the same nutritional profile. Wild meat and game that could be acquired through hunting may provide higher levels of essential fatty acids and protein [63, 64], which could provide dietary quality benefits.

These findings may have important long-term health implications, especially the finding that nearly one in four respondents was eating less fruits and vegetables during the COVID-19 pandemic than before. Increased fruit and vegetable intake is associated with reduced risk of cardiovascular disease, certain cancers, and all-cause mortality [65], yet even pre-pandemic, most Americans did not meet the national recommendations for fruit and vegetable intake [66]. Our finding of reduced intake are similar to those from studies conducted recently in France [38] and the United Arab Emirates [37] finding lower fruit and vegetable intake during COVID-19 associated lockdowns. Respondents using HFP were on average eating ½ cup more of fruits and vegetables daily; higher fruit and vegetable intake is associated with reduced risk of cardiovascular disease, cancer and mortality [65]. Furthermore, since previous research suggests that gardening is also associated with improved nutritional knowledge [33, 34], and long-term beneficial changes in eating habits [24, 35], the significant uptick in gardening and other HFP strategies during the pandemic may have future impacts on diet quality and health not yet realized. Future research should continue to monitor these potential changes, including their link to health outcomes more specifically.

There are many opportunities to expand this work with future research and address potential limitations of the current study. One limitation of this study is a lack of understanding about the amount of food generated through HFP activities. Future research could more clearly explore how different quantities of HFP affect food security and diet quality outcomes by asking what percent of food intake is coming from HFP, or whether HFP activities, especially hunting, fishing and foraging, reliably result in food procurement. Second, future analyses would benefit from more nuanced and complete measurement of dietary intake, including measurement of white meat, fish, and seafood, as these are nutrient-dense foods that may be acquired through HFP. A limitation of the present study was the measurement of only fruit, vegetable, red meat, and processed meat intake (selected for their strong associations to diet-related disease outcomes), rather than a broader portfolio of foods and nutrients. Further, in some of our diet quality metrics, we combined red and processed meat, which may have different nutritional profiles, especially if wild meat is part of a diet. These should be more carefully separated in future studies. Third, our work includes self-reports of dietary intake, which are known to have limitations in their accuracy (especially for energy intake, which we do not assess here), but are still the primary way in which dietary intake data is collected and continue to be recommended for use [67]. Indeed, while we utilized a sampling strategy that would be at least partially representative of characteristics of the state, response bias in questions may still be possible with our data. Fourth, this work demonstrates outcomes during a global pandemic, when many people’s daily lives were significantly changed. People potentially had new motivations for pursuing HFP activities that could be related to food security, but also may be unrelated (e.g. hobbies, time in nature, cultural trends). Long-term potential diet and food security costs and benefits from HFP will likely accrue over many years. Therefore, it is critical to assess whether the new and increased intensity of HFP is sustained in the future. Such sustained efforts would also potentially have important impacts on conservation through increased demand in hunting, fishing and foraging that should be adequately considered. As well, long-term increased engagement in HFP activities may require increased resources for people pursuing these activities, which could happen through educational efforts, cost-share or grants for infrastructure (e.g. garden beds) and equipment (e.g. tools), especially since gardening can have significant up-front costs [26]. Fifth, our study population is from a predominantly rural state, which may influence the ability of people to access land for engaging in HFP. In more urban settings, access to land for gardening, or ability to engage in other HFP may be more limited [68, 69], especially if residents need to travel significant distances [70, 71]. Finally, given the social distanced nature of COVID-19, this research utilized an online survey to capture an understanding of this issue, but this research would certainly benefit from additional qualitative and quantitative data analysis. Interviews and focus groups could contextualize the results and better understand the motivations and challenges of HFP activities, which can provide important information for future education and resource allocation. Future studies would benefit from a longitudinal or interventional design that support the examination of causality.

## Conclusion

This study documented the extent of a range of HFP activities among a statewide sample in the US and assessed associations between HFP and food security and dietary outcomes. The results demonstrate that HFP activities significantly increased during the first five months of the COVID-19 pandemic, and were especially prominent among food insecure households. The results also document clear relationships between HFP activities and dietary outcomes, including higher fruit and vegetable intake, which may have important health benefits long-term. Taken together, the results suggest that HFP activities are an important, and potentially increasingly important, way in which many people engage in the food system and the natural environment, with potential implications for both conservation and nutrition and health outcomes. As such, additional research should aim to more fully understand these relationships over time, and in greater depth, especially in the continuation and aftermath of the COVID-19 pandemic. As well, additional collaborations within the conservation sector may be important to assess the long-term impact of increased levels of HFP that may affect forests, waterways, and species. Heightened engagement in HFP may necessitate expanded education and outreach efforts to provide resources for HFP that is productive and sustainable.

## Supplementary Information

**Additional file 1.**

## Data Availability

The survey instrument materials used for this current study are available at Harvard Dataverse at: https://dataverse.harvard.edu/dataverse/foodaccessandcoronavirus . The datasets used and/or analyzed during the current study are available from the corresponding author on reasonable request.
