# Supplementary material for: Home food procurement impacts food security and diet quality during COVID-19
Source: BMC Public Health. 2021 May 19;21:945. doi: 10.1186/s12889-021-10960-0 (PMC8131171; doi:10.1186/s12889-021-10960-0)
Supplement: Supplementary file 1 — Additional file 1. [file 12889_2021_10960_MOESM1_ESM.docx]

**Supplementary Materials**

**Home Food Procurement Impacts Food Security and Diet Quality during COVID-19**

Meredith T. Niles, Kristen Brassard Wirkkala, Emily H. Belarmino, Farryl Bertmann

Supplementary Table 1. Results of a logit model predicting food security with demographic controls.

| **Variable Name** | **Odds Ratio** | **Std. Err.** | **p=** | **95% Confidence Interval** | |
| --- | --- | --- | --- | --- | --- |
| Female | 1.052 | 0.261 | 0.840 | 0.646 | 1.710 |
| Children in HH | 0.842 | 0.253 | 0.567 | 0.467 | 1.518 |
| Over 55 | 2.518 | 0.674 | 0.001 | 1.490 | 4.256 |
| BIPOC/Hispanic | 1.606 | 0.663 | 0.251 | 0.715 | 3.605 |
| Negative Job Change | 0.474 | 0.106 | 0.001 | 0.306 | 0.733 |
| Less50k | 0.134 | 0.031 | 0.000 | 0.085 | 0.211 |
| HH Size | 0.857 | 0.089 | 0.139 | 0.699 | 1.051 |

Supplementary Table 2. Food insecurity by disaggregated race and ethnicity.

|  | Food Security Rate | |  |  |
| --- | --- | --- | --- | --- |
|  | For Demographic Group | For Outside Demographic Group | Total in Demographic Group | p= (chi2 test) |
| Asian | 75.0% | 70.9% | 4 | 0.858 |
| Black | 50.0% | 71.3% | 8 | 0.188 |
| Native American | 80.0% | 70.9% | 5 | 0.655 |
| Multiple Race | 66.6% | 71.1% | 21 | 0.066 |
| White | 71.3% | 65.8% | 544 | 0.467 |
| BIPOC/Hispanic | 63.8% | 71.6% | 47 | 0.261 |
| Hispanic | 50.0% | 71.6% | 16 | 0.061 |

Supplementary Table 3. Logit model predicting gardening activity since COVID-19 by demographic controls.

| **Variable** | **Odds Ratio** | **Std. Error** | **p=** | **95% Confidence Interval** | |
| --- | --- | --- | --- | --- | --- |
| Female | 0.905 | 0.176 | 0.606 | 0.618 | 1.324 |
| Children in HH | 1.341 | 0.360 | 0.274 | 0.793 | 2.269 |
| Over 55 | 1.351 | 0.291 | 0.162 | 0.886 | 2.060 |
| BIPOC/Hispanic | 0.884 | 0.295 | 0.711 | 0.459 | 1.700 |
| Negative Job Change | 1.425 | 0.263 | 0.055 | 0.993 | 2.047 |
| Less $50K | 0.632 | 0.118 | 0.014 | 0.438 | 0.912 |
| HH Size | 0.867 | 0.080 | 0.124 | 0.723 | 1.040 |

Supplementary Table 4. Logit model predicting fishing activity since COVID-19 by demographic controls.

| **Variable** | **Odds Ratio** | **Std. Error** | **p=** | **95% Confidence Interval** | |
| --- | --- | --- | --- | --- | --- |
| Female | 0.732 | 0.220 | 0.299 | 0.407 | 1.318 |
| Children in HH | 1.265 | 0.484 | 0.539 | 0.598 | 2.677 |
| Over 55 | 0.501 | 0.177 | 0.051 | 0.250 | 1.003 |
| BIPOC/Hispanic | 1.095 | 0.517 | 0.848 | 0.434 | 2.763 |
| Negative Job Change | 1.550 | 0.449 | 0.131 | 0.878 | 2.735 |
| Less $50K | 0.718 | 0.211 | 0.258 | 0.404 | 1.276 |
| HH Size | 0.939 | 0.125 | 0.634 | 0.723 | 1.218 |

Supplementary Table 5. Logit model predicting foraging activity since COVID-19 by demographic controls.

| **Variable** | **Odds Ratio** | **Std. Error** | **p=** | **95% Confidence Interval** | |
| --- | --- | --- | --- | --- | --- |
| Female | 1.207 | 0.397 | 0.567 | 0.633 | 2.302 |
| Children in HH | 1.260 | 0.535 | 0.586 | 0.549 | 2.895 |
| Over 55 | 1.846 | 0.682 | 0.097 | 0.894 | 3.809 |
| BIPOC/Hispanic | 1.162 | 0.594 | 0.769 | 0.427 | 3.162 |
| Negative Job Change | 2.130 | 0.652 | 0.014 | 1.169 | 3.881 |
| Less $50K | 1.463 | 0.438 | 0.204 | 0.813 | 2.630 |
| HH Size | 1.119 | 0.158 | 0.427 | 0.848 | 1.477 |

Supplementary Table 6. Logit model predicting hunting activity since COVID-19 by demographic controls.

| **Variable** | **Odds Ratio** | **Std. Error** | **p=** | **95% Confidence Interval** | |
| --- | --- | --- | --- | --- | --- |
| Female | 0.458 | 0.169 | 0.034 | 0.222 | 0.942 |
| Children in HH | 2.263 | 1.105 | 0.094 | 0.870 | 5.890 |
| Over 55 | 0.594 | 0.277 | 0.265 | 0.238 | 1.483 |
| BIPOC/Hispanic | 1.529 | 0.812 | 0.424 | 0.540 | 4.328 |
| Negative Job Change | 1.642 | 0.614 | 0.185 | 0.789 | 3.416 |
| Less $50K | 0.900 | 0.333 | 0.777 | 0.436 | 1.859 |
| HH Size | 0.836 | 0.144 | 0.299 | 0.597 | 1.172 |

Supplementary Table 7. Logit model predicting backyard livestock activity since COVID-19 by demographic controls.

| **Variable** | **Odds Ratio** | **Std. Error** | **p=** | **95% Confidence Interval** | |
| --- | --- | --- | --- | --- | --- |
| Female | 0.721 | 0.276 | 0.392 | 0.340 | 1.526 |
| Children in HH | 1.248 | 0.560 | 0.621 | 0.518 | 3.005 |
| Over 55 | 0.157 | 0.091 | 0.001 | 0.050 | 0.491 |
| BIPOC/Hispanic | 1.404 | 0.740 | 0.519 | 0.500 | 3.944 |
| Negative Job Change | 1.487 | 0.551 | 0.285 | 0.719 | 3.075 |
| Less $50K | 0.741 | 0.271 | 0.412 | 0.362 | 1.518 |
| HH Size | 0.962 | 0.149 | 0.800 | 0.710 | 1.303 |

Supplementary Table 8. Logit model predicting canning activity since COVID-19 by demographic controls.

| **Variable** | **Odds Ratio** | **Std. Error** | **p=** | **95% Confidence Interval** | |
| --- | --- | --- | --- | --- | --- |
| Female | 0.920 | 0.198 | 0.698 | 0.604 | 1.402 |
| Children in HH | 1.342 | 0.395 | 0.318 | 0.754 | 2.388 |
| Over 55 | 1.317 | 0.321 | 0.257 | 0.818 | 2.122 |
| BIPOC/Hispanic | 1.417 | 0.485 | 0.309 | 0.724 | 2.773 |
| Negative Job Change | 1.454 | 0.297 | 0.067 | 0.973 | 2.171 |
| Less $50K | 0.703 | 0.147 | 0.091 | 0.467 | 1.058 |
| HH Size | 0.963 | 0.097 | 0.704 | 0.790 | 1.172 |

Supplementary Table 9. Percent of Respondents by Food Security Status Engaging in HFP activities. P values determined through chi-square tests.

| **Activity** | **Food Secure** | **Food Insecure** | **p=** |
| --- | --- | --- | --- |
| Home Food Procurement | 33.4% | 35.9% | 0.564 |
| More HFP since COVID | 44.4% | 66.2% | 0.002 |
| Gardens Since | 34.3% | 35.5% | 0.782 |
| Fishing Since | 7.7% | 15.4% | 0.005 |
| Foraging Since | 7.0% | 14.8% | 0.003 |
| Hunting Since | 3.9% | 11.8% | 0.000 |
| Livestock Since | 4.3% | 10.1% | 0.008 |
| Canning Since | 20.5% | 29.6% | 0.019 |
| Gardens More | 38.8% | 58.9% | 0.005 |
| Fishing More | 28.3% | 51.1% | 0.025 |
| Foraging More | 34.3% | 59.4% | 0.040 |
| Hunting More | 14.3% | 47.2% | 0.003 |
| Livestock More | 47.6% | 60.0% | 0.401 |
| Canning More | 53.1% | 63.3% | 0.136 |

Supplementary Table 10. Matching results examining current fruit intake, with various treatment variables. Each row indicate a separate matching result.

| **Variable** | **Coefficient** | **Robust Std. Error** | **p=** | **95% Confidence Interval** | | **Treated n= (Matched n=)** | **Control n= (Matched n)** |
| --- | --- | --- | --- | --- | --- | --- | --- |
| HFP | 0.292 | 0.125 | 0.019 | 0.047 | 0.537 | 201 (201) | 364 (201) |
| HFP More | -0.060 | 0.252 | 0.811 | -0.554 | 0.433 | 123 (123) | 117 (123) |
| Garden Since | 0.392 | 0.120 | 0.001 | 0.157 | 0.627 | 203 (203 | 368 (203) |
| Foraging Since | 0.154 | 0.225 | 0.493 | -0.287 | 0.596 | 54 (54) | 517 (54) |
| Canning Since | 0.275 | 0.136 | 0.044 | 0.008 | 0.542 | 139 (139) | 432 (139) |
| Gardens More | -0.048 | 0.210 | 0.821 | -0.459 | 0.364 | 102 (102) | 125 (102) |
| Foraging More | 0.109 | 0.706 | 0.877 | -1.274 | 1.493 | 30 (30) | 37 (30) |
| Canning More | 0.345 | 0.335 | 0.302 | -0.311 | 1.001 | 61 (61) | 94 (61) |

Supplementary Table 11. Matching results examining current vegetable intake, with various treatment variables. Each row indicates a separate matching result.

| **Variable** | **Coefficient** | **Robust Std. Error** | **p=** | **95% Confidence Interval** | | **Treated n= (Matched n=)** | **Control n= (Matched n)** |
| --- | --- | --- | --- | --- | --- | --- | --- |
| HFP | 0.487 | 0.124 | 0.000 | 0.244 | 0.730 | 201 (201) | 364 (201) |
| HFP More | -0.132 | 0.204 | 0.516 | -0.531 | 0.267 | 123 (123) | 117 (123) |
| Garden Since | 0.551 | 0.122 | 0.000 | 0.313 | 0.790 | 203 (203) | 368 (203) |
| Foraging Since | 0.260 | 0.201 | 0.196 | -0.134 | 0.653 | 54 (54) | 517 (54) |
| Canning Since | 0.513 | 0.142 | 0.000 | 0.234 | 0.791 | 139 (139) | 432 (139) |
| Gardens More | -0.020 | 0.178 | 0.909 | -0.368 | 0.327 | 102 (102) | 125 (102) |
| Foraging More | 0.048 | 0.665 | 0.942 | -1.255 | 1.352 | 30 (30) | 37 (30) |
| Canning More | -0.121 | 0.386 | 0.754 | -0.877 | 0.635 | 61 (61) | 94 (61) |

Supplementary Table 12. Matching results examining current red meat intake, with various treatment variables. Each row indicates a separate matching result.

| **Variable** | **Coefficient** | **Robust Std. Error** | **p=** | **95% Confidence Interval** | | **Treated n= (Matched n=)** | **Control n= (Matched n)** |
| --- | --- | --- | --- | --- | --- | --- | --- |
| HFP | 0.038 | 0.174 | 0.828 | -0.303 | 0.379 | 201 (201) | 364 (201) |
| HFP More | -0.083 | 0.273 | 0.762 | -0.618 | 0.452 | 123 (123) | 117 (123) |
| Fishing Since | 0.275 | 0.265 | 0.301 | -0.246 | 0.795 | 59 (59) | 512 (59) |
| Hunting Since | 0.325 | 0.275 | 0.238 | -0.215 | 0.865 | 35 (35) | 536 (35) |
| Livestock Since | 1.020 | 0.314 | 0.001 | 0.404 | 1.635 | 37 (37) | 534 (37) |
| Fishing More | -0.204 | 0.746 | 0.784 | -1.667 | 1.258 | 38 (38) | 55 (38) |
| Hunting More | -0.662 | 0.499 | 0.184 | -1.639 | 0.315 | 20 (20) | 51 (20) |
| Livestock More | -0.148 | 0.509 | 0.772 | -1.145 | 0.850 | 26 (26) | 23 (26) |

Supplementary Table 13. Matching results examining current processed meat intake, with various treatment variables. Each row indicates a separate matching result.

| **Variable** | **Coefficient** | **Robust Std. Error** | **p=** | **95% Confidence Interval** | | **Treated n= (Matched n=)** | **Control n= (Matched n)** |
| --- | --- | --- | --- | --- | --- | --- | --- |
| HFP | -0.365 | 0.163 | 0.025 | -0.685 | -0.046 | 201 (201) | 364 (201) |
| HFP More | 0.180 | 0.274 | 0.512 | -0.357 | 0.717 | 123 (123) | 117 (123) |
| Fishing Since | 0.218 | 0.268 | 0.416 | -0.308 | 0.744 | 59 (59) | 512 (59) |
| Hunting Since | -0.111 | 0.363 | 0.760 | -0.821 | 0.600 | 35 (35) | 536 (35) |
| Livestock Since | 0.352 | 0.332 | 0.289 | -0.298 | 1.002 | 37 (37) | 534 (37) |
| Fishing More | 0.761 | 0.499 | 0.217 | -0.217 | 1.738 | 38 (38) | 55 (38) |
| Hunting More | -0.065 | 0.555 | 0.906 | -1.153 | 1.022 | 20 (20) | 51 (20) |
| Livestock More | 0.201 | 0.487 | 0.666 | -0.745 | 1.165 | 26 (26) | 23 (26) |

Supplementary Table 14. Matching results examining change in fruit and vegetable intake since COVID-19, with various treatment variables. Each row indicates a separate matching result.

| **Variable** | **Coefficient** | **Robust Std. Error** | **p=** | **95% Confidence Interval** | | **Treated n= (Matched n=)** | **Control n= (Matched n)** |
| --- | --- | --- | --- | --- | --- | --- | --- |
| HFP | 0.116 | 0.053 | 0.029 | 0.012 | 0.220 | 201 (201) | 364 (201) |
| HFP More | -0.047 | 0.119 | 0.696 | -0.280 | 0.187 | 123 (123) | 117 (123) |
| Garden Since | 0.079 | 0.051 | 0.124 | -0.022 | 0.179 | 203 (203) | 368 (203) |
| Foraging Since | 0.052 | 0.095 | 0.580 | -0.134 | 0.239 | 54 (54) | 517 (54) |
| Canning Since | 0.119 | 0.061 | 0.051 | -0.001 | 0.239 | 139 (139) | 432 (139) |
| Gardens More | -0.030 | 0.101 | 0.765 | -0.228 | 0.168 | 102 (102) | 125 (102) |
| Foraging More | -0.157 | 0.217 | 0.470 | -0.581 | 0.268 | 30 (30) | 37 (30) |
| Canning More | -0.043 | 0.206 | 0.834 | -0.447 | 0.361 | 61 (61) | 94 (61) |

Supplementary Table 15. Matching results examining change in red and processed meat intake since COVID-19, with various treatment variables. Each row indicates a separate matching result.

| **Meat Change** | **Coefficient** | **Robust Std. Error** | **p=** | **95% Confidence Interval** | | **Treated n= (Matched n=)** | **Control n= (Matched n)** |
| --- | --- | --- | --- | --- | --- | --- | --- |
| HFP | -0.024 | 0.057 | 0.678 | -0.135 | 0.088 | 200 (200) | 364 (200) |
| HFP More | -0.120 | 0.110 | 0.276 | -0.336 | 0.096 | 122 (122) | 117 (122) |
| Fishing Since | 0.051 | 0.091 | 0.576 | -0.128 | 0.230 | 59 (59) | 511 (59) |
| Hunting Since | 0.077 | 0.110 | 0.485 | -0.139 | 0.293 | 35 (35) | 535 (35) |
| Livestock Since | 0.042 | 0.112 | 0.707 | -0.177 | 0.262 | 37 (37) | 533 (37) |
| Fishing More | -0.152 | 0.233 | 0.515 | -0.609 | 0.305 | 38 (38) | 55 (38) |
| Hunting More | -0.104 | 0.188 | 0.581 | -0.473 | 0.265 | 20 (20) | 51 (20) |
| Livestock More | -0.056 | 0.154 | 0.717 | -0.358 | 0.246 | 26 (26) | 23 (26) |
